# Assessment of post-harvest losses and carbon footprint in intensive lowland rice production in Myanmar

**DOI:** 10.1038/s41598-020-76639-5

**Published:** 2020-11-13

**Authors:** Martin Gummert, Nguyen-Van-Hung, Christopher Cabardo, Reianne Quilloy, Yan Lin Aung, Aung Myo Thant, Myo Aung Kyaw, Romeo Labios, Nyo Me Htwe, Grant R. Singleton

**Affiliations:** 1 International Rice Research Institute, 4031 Los Banos, Laguna Philippines; 2 Southeast Asian Regional Center for Graduate Study and Research in Agriculture, 4031 Los Banos, Laguna Philippines; 3 Department of Agriculture, Nay Pyi Taw, Myanmar; 4 Natural Resources Institute, University of Greenwich, Chatham Maritime, Kent UK

**Keywords:** Ecology, Environmental sciences, Engineering

## Abstract

This paper examines how a move from traditional post-harvest operations of smallholder rice farms in the Ayeyarwaddy delta, Myanmar, to improved post-harvest operations affected income, energy efficiency and greenhouse gas emissions (GHGE). Harvest and post-harvest losses were investigated in a field experiment with 5 replications per scenario. A comparative analysis on energy efficiency and cost-benefits was conducted for different practices of rice production from cultivation to milling. GHGE of different practices were also considered using a life-cycle assessment approach. The study demonstrates that the mechanized practices increased the net income by 30–50% compared with traditional practices. Despite using additional energy for machine manufacturing and fuel consumption, the mechanized practices significantly reduced postharvest losses and did not increase the total life-cycle enegy and GHGE. Combine harvesting helped to significantly reduce harvesting loss in a range of 3 to 7% (by weight of the rice product). Improved post-harvest management practices with a flatbed dryer and hermetic storage reduced the discoloration of rice grains by 3 to 4% and increased head-rice recovery by 20 to 30% (by weight of rice product). The research findings provide empirical evidence that improved post-harvest management of rice in the Ayeyarwaddy delta, compared to traditional post-harvest operations by smallholder farmers, reduce post-harvest losses and improve the quality of rice. The findings provide valuable information for policy makers involved in formulating evidence-based mechanization policies in South and Southeast Asia.

## Introduction

Approximately 500 million tons of milled rice per year are produced globally, of which 90% come from Asian countries^1^. Increased grain quality and productivity, and reduction of losses are key targets for improved post-harvest management of rice production for smallholder farmers in Asia. Rice post-production processes, which involves harvesting to milling, are estimated to incur losses of 20–30% of rice grain produced. However, there are limited quantitative data available; most are reports based on qualitative surveys such as by the Food and Agriculture Organization of the United Nations (FAO)^2^ and the International Rice Research Institute (IRRI)^3^. There is a wide range of post-harvest management practices for rice applied across Asia. For instance, mechanized post-harvest operations are uncommon in Myanmar^4^, but are common in Thailand^5^ and Vietnam^6^. One of the major constraints for rice production in Myanmar is low level of mechanization^4^, which is also considered a key factor to increase efficiency of rice production^7^.

Moving from traditional post-harvest operations in developing nations to mechanized systems adds considerable investment costs, particularly for machinery and may also lead to increased Greenhouse Gas Emissions (GHGE) from machine production and fuel consumption^6–8^. However, post-harvest losses under traditional operations may be considerably higher compared to mechanized systems. For example, in rice–pulse systems in the Ayeyarwady delta of Myanmar, farmers manually harvest their monsoon rice crop and then often stack the cut crop in the field or on bunds for 1 to 4 weeks until labor and equipment is available for the threshing process. Farmers adopt this practice to take advantage of the residual soil moisture content to establish a pulse crop^9^. Delayed threshing of rice causes high quantitative (e.g. shattering, loss to birds, and rodents) and qualitative losses (discoloration, mouldy and broken grains)^10^.

There are two main harvesting practices for rice in Southeast Asia (SEA): (1) manual cutting and mechanical threshing; and (2) using combine harvesters^8^. After manual cutting, the harvest operation is often delayed because of labor shortage during the harvest period. Furthermore, freshly cut rice plants are often stacked in the field to dry before threshing because the available machines cannot handle wet rice plants. Delay of harvesting or in-field stacking of rice plants may cause high losses due to shattering, consumption by rodents^9^, damage from insects and moulds, and fissuring of grains and discoloration^11^. In tropical countries, paddy is usually harvested at 20–28% grain moisture content wet basic (MCwb) depending on whether it is the wet or dry season^12^.

In Southeast Asia, sun drying and flatbed dryers are the two main methods for drying of rice grain^6^. Sun drying often causes high losses of both quantity and quality due to exposure of the grain to weather, rodents, and birds that are largely resolved by using mechanical dryers such as a flatbed dryer^6,13^. Paddy storage is also a key operation that in developing countries often contributes to losses in rice value chains. Poor storage causes high losses due to pests^9^.

The economic and technical efficiency of post-harvest systems in rice production have been quantified in Korea^14^, Bangladesh^15^, Pakistan^16^, Thailand^17^, Myanmar^4^, and Vietnam^18^. Quantification of energy efficiency and GHGE of rice production using a life cycle assessment (LCA) approach has been reported in a global database^19^ and in specific studies in different countries such as the Philippines^20^, Japan^21^, Iran^22^, Italy^23^, and Canada^24^. Post-harvest losses and mechanization also have been investigated for specific processes such as farm mechanization^25^, drying^6^, and mechanized rice straw collection^26^.

However, there is no published synthesis that compares different post-harvest management practices to identify best options for minimizing losses, production cost, and the environmental footprint of post-harvest practices. The objectives of the study in Myanmar were to: (i) assess the energy and costs for the production of grain in traditional post-harvest systems, (ii) assess the GHGE incurred by using mechanized post-harvest options, and (iii) compare the GHGE, energy and costs incurred under both scenarios.

## Methods

### Research scope and experimental design

**Figure 1:**
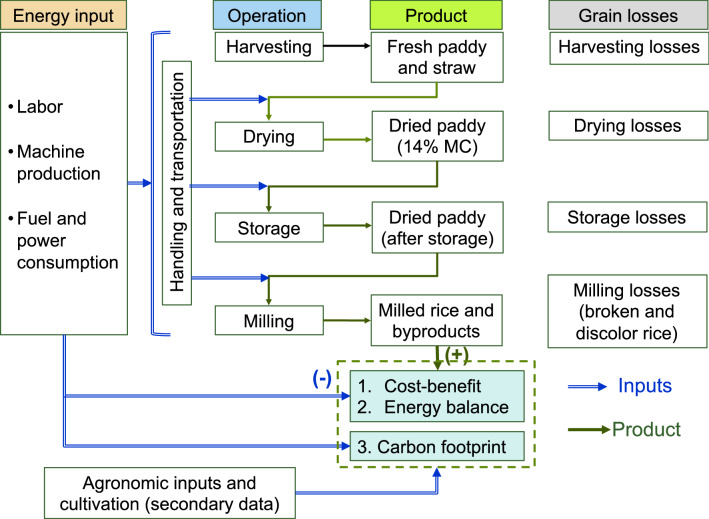
Inputs and outputs of the research system.

The study was conducted on rice production in Tar Pat Village, Maubin, Myanmar (16.617° N, 95.680° E) in the wet season (WS) 2014 and the dry seasons (DS) of 2015 and 2016. Sin Thukha variety with a growing time of 135 days was used for all 3 seasons. Best practices were identified based on the indicators of energy balance, cost-benefits, and GHGE for a functional unit (FU) of 1 ha of rice production. The last factor (GHGE) was estimated using the attributional LCA^27–29^ approach following LCA ISO standard ISO1404:44. Figure 1 shows the system boundary covering all processes of rice production from preharvest (cultivation) to postharvest (until milling). The primary data were collected in harvest and postharvest processes while the secondary data of pre-harvest processes were used in the system analysis. The conversion factors for energy and GHGE of the agronomic inputs, fuel and power consumption, and related transportations were interpreted from the ECOINVENT 3 database (version 3.3)^19^.

Table 1 shows the research treatments with their major features and applied practices in the different seasons. For the WS2014, a comparative analysis was conducted for two farmer practices (FPs) and one improved practice (IPR). The two farmer practices corresponded to the scenarios of stacking rice plants in the field for 1 week (FP1w) and for 4 weeks (FP4w) after manual cutting. The IPR scenario involved threshing within 12 h after harvest. In addition, the IPR included use of a flatbed dryer for drying the rice, and hermetic bags for storage, instead of sun drying and farmer-granary bags for storage under FP.

**Table 1:** Scenarios and post-harvest operations covered in the study in Maubin, Ayeyarwady delta, Myanmar during three rice cropping seasons.

Scenarios	Post-harvest operations				
	Harvesting		Drying	Storage	Milling
	Cutting	Threshing			
**WS2014**					
FP1w: Farmer practice with 1-week delay of threshing	Manual cutting, stacking rice 1 week in field s	Farmer thresher	Sun drying	Granary bag	Using local rice mill 1 t h^−1^ for all treatments
FP4w: Farmer practice with 4 weeks delay of threshing	Manual cutting, stacking rice 4 weeks in fields				
IPR: Improved post-harvest practice with improved thresher, flatbed dryer, and hermetic storage	Manual cutting, threshing immediately after cutting	Improved thresher	Flatbed dryer	Hermetic Super bag	
**DS2015 and 2016**					
FP: farmer practice	Manual cutting	Farmer thresher	Sun drying	Granary bag	Using local rice mill 1 t h^−1^ for all treatments
IPRc: Improved post-harvest practice with combine harvester, flatbed dryer, and hermetic storage	Combine harvester		Flatbed dryer	Hermetic Super bag	

In the dry season of 2015 and 2016, the analysis was conducted for two scenarios, which were farmer practice (FP) and improved post-harvest operations with a combine harvester, flatbed dryer, and hermetic storage (IPRc). Neither of the scenarios had delays or stacking of the rice plants, because farmers were able to thresh the rice immediately after it was manually harvested. The practices involved in FP were manual operations such as cutting of the mature rice plants and sun-drying. The scenarios of DS2015 and DS2016 differed from the WS2014 because the farmers did not stack rice in the DS prior to threshing.

**Figure 2:**
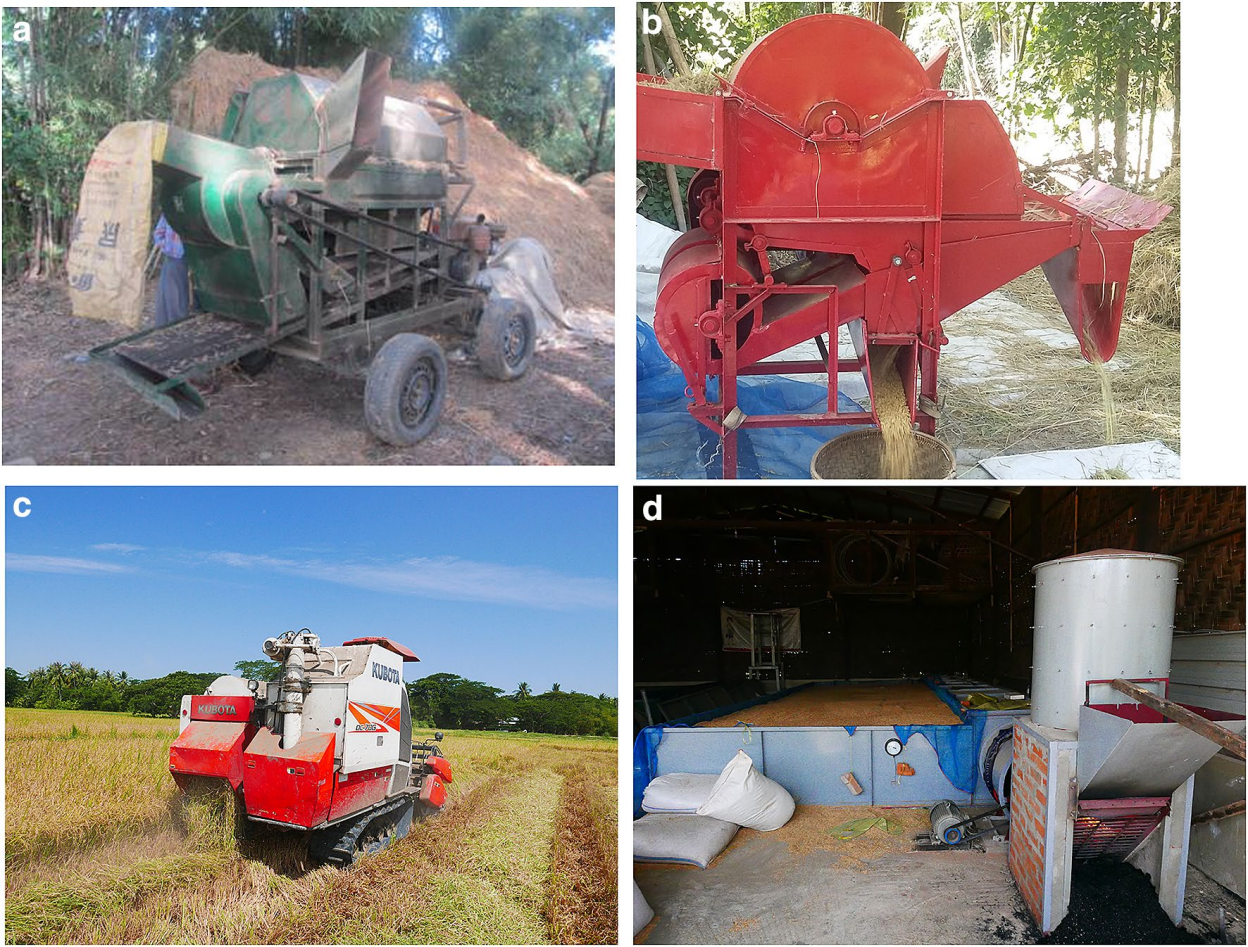
(**a**) Farmer thresher. (**b**) Imported thresher, TC-800. (**c**) Combine harvester Kubota-DC-70G. (**d**) Flatbed dryer 4 ton batch^−1^.

The experiment was set up in fields of farmers on 4110 m^2^ for WS2014 and 5850 m^2^ for both DS2015 and DS2016. Each scenario was replicated 5 times in the different plots and were distributed using a completely randomized design (CRD). For the WS2014 experiment, there were 15 plots for three scenarios with each plot 270 m^2^. The paddy was harvested and processed based on the respective IPR and FP scenarios. The FP scenario included a thresher locally fabricated by the farmers based on the axial threshing principle that was powered by a two-wheel tractor with a 15 HP diesel engine (Fig. 2a), sun drying, and granary bags containing approximately 50 kg of paddy each. The IPR scenario involved a TC-800 axial flow thresher with a 7.5 HP engine (Fig. 2b)^30^. Compared to the farmer thresher, the imported unit was manufactured and marketed by a branded company in the Philippines and tested by IRRI to ensure good performance. For DS2015 and DS2016, there were 10 plots for two scenarios with each plot 390 m^2^. Rice was harvested using a Kubota-DC-70G combine harvester with 70 HP (Fig. 2c). A flatbed dryer and hermetic bags for storage of dried grain were used for IPR in all three seasons. The flatbed dryer with 4 t batch^−1^ capacity (Fig. 2d) was locally made based on published designs^6^, and was used for the IPR in both the wet and dry seasons. Hermetic bags for storage, also called “Super bags”^31^ hold 50 kg of paddy per bag. Milling operations were in-situ measured at the local rice mill (two-stage milling system) with 1 t h^−1^ capacity, located at Maubin, and were applied for both FP and IPR scenarios.

### Measurement and quantification of harvest and post-harvest losses

Shattering loss during cutting, stacking, and combine harvesting was determined through sampling of 5 plots using 1 m^2^ quadrants for each scenario. Shattering losses were calculated based on the ratio between shattered grains and yield at an adjusted moisture content of 14% wet basis (MC). In threshing, the grain losses were quantified in the stacked rice, at the separation process, in the cleaning process, and under the machine during the threshing operation. The sum of these losses comprised the threshing loss. The design did not quantify losses caused in drying and storage during handling, and grain lost to birds and rodents. See Htwe et al.^9^ for estimate of losses caused by rodents at this study site. The losses associated with discoloration, milling recovery (MR), and head rice recovery (HRR) caused by in-field stacking, delay of drying and storage methods were measured after milling.

MR and HRR were measured on milled rice. Three subsamples of 500 g of paddy were taken randomly from the grain harvested in each plot. The samples were cleaned using a Seedburo Paddy Blower, then 250 g of filled grain were passed twice in a RISE 10″ Rubber Roll Husker, then through a SATAKE Abrasive Whitener, and finally, through a SATAKE laboratory rice grader. MR and HRR were calculated using Eqs. (1) and (2), respectively.

1 $$MR\; (\%)=\frac{Weight\;of\;milled \;rice \;\left(include \;broken\; grains\right)}{Weight\; of \;paddy \;samples} \times100$$

2 $$HRR \;(\%) =\frac{Weight\; of \;whole\; grains}{Weight \;of\; paddy \;samples} \times 100$$

Discoloration of grain was caused by fungi, bacteria, and environmental conditions such as high humidity and temperature. Milled rice kernels having more than 0.5% grains with a color other than white (usually yellow) or with a spotted surface were considered discolored^32^. To measure discoloration, three 25 g samples of the product were collected randomly. Discolored grains with spots, streaks, or having more than 0.5% differently colored surface were separated and weighed to calculate the percentage of discoloration based on Eq. (3).

3 $$Discoloration \;(\%)= \frac{weight \;of \;discolored \;grains (\text{g})}{weight \;of \;sample\; (25 \;\text{g})} \times 100$$

### Energy efficiency and GHG emissions

This study investigated net energy value (NEV) and net energy ratio (NER) which are commonly used to quantify energy efficiency of a production systems^33–35^. NEV accounted for the inputs and outputs of the systems per the FU (ha of rice production) (Eq. 4) while the NER was the ratio between the output and input energy values (Eq. 5).

4 $$NEV \left(\frac{GJ}{ha}\right)=E{V}_{outputs}-E{V}_{inputs}$$

where the *EV*_*outputs*_ accounted for rice grain products and by-products such as broken and discolored grains, bran, husks, and straw. The *EV*_*inputs*_ includes all the energy consumption of rice production from cultivation to milling; this includes agronomic inputs, machine production, fuel and power consumption, and labor use.

5 $$NER=\frac{E{V}_{outputs}}{E{V}_{inputs}}$$

Table 2 shows energy values embed in the whole milled rice, broken and discolored rice, bran, husk, and straw. Energy value (EV) of rice product^36^ is 15.2 MJ kg^−1^ while that of rice bran, broken rice, and discolored rice is 9.6 MJ kg^−1^ (Econivent 3 database^19^) with an assumption that these by-products are used for cattle feed and have a similar economic value. EV of rice husk is 8.7 MJ kg^−1^ (Ecoinvent 3 database^19^) and straw is 3.5 MJ kg^−1^^20^ based on an asumption that partially harvested straw were collected for mushroom production. The collected amount of rice straw was about 50% of the grain yield at harvest^34^. The EV per kg was then translated to the FU based on the grain yield and post-harvest losses measured in the experiments. In particular, rice husk and bran were assumed to be 20 and 10% of the milled rice produced, respectively. EV of the cultivation (excluding harvesting and transportation) was about 12 and 16 GJ ha^−1^ for the small-farm irrigated rice production in the WS and DS, respectively, as reported in research in the same region (Ayeyarwaddy delta of Myanmar)^37^. EV of machine production was accounted for via a depreciation of 5 years. Fuel and power consumption of harvest and post-harvest operations were measured and translated to EV using the coversion factors. The energy of manual labor was calculated based on the metabolic equivalent of tasks (MET) (Table 2). Ainsworth et al.^38^ described the MET as the ratio of the human metabolic rate when performing an activity to the metabolic rate at rest. This ratio is converted to an energy value as MJ per hour working using the method described by Quilty et al.^39^ with the assumption of a mean Asian human body weight of 55 kg. For paddy transportation, the tractor-hauled trailor option was used for all scenarios with a distance of 15 km from the field to the station of drying, storage, and milling.

**Table 2:** Conversion factors for energy and GHGE.

Parameters	Energy			GHGE		
	Unit	Value	Reference	Unit	Value	Reference
Rice cultivation in WS	GJ ha^−1^	12	^37^	MgCO_2_-eq ha^1^	2.0	^40^
Rice cultivation in DS	GJ ha^−1^	16	^37^	MgCO_2_-eq ha^1^	1.2	^40^
Diesel consumption	MJ L^−1^	44.8	^19^	kgCO_2_-eq MJ^−1^	0.08	^19^
Machine production^a^	MJ L^−1^	15.6	^44, 45^			
Electric power (including motor production)	MJ kWh^−1^	10.7	^19^	kgCO_2_-eq kWh^−1^	0.615	^19^
Transporting paddy	MJ tkm^−1^	5.66	^19^	kgCO_2_-eq tkm^−1^	0.41	^19^
Producing polyethylene net and plastic bags	MJ kg^−1^	78.2	^19^	kgCO_2_-eq kg^−1^	1.8	^19^
**Materials to produce the flatbed dryer**						
Brick	MJ kg^−1^	2.69	^19^	kgCO_2_-eq kg^−1^	0.251	^19^
Cement	MJ kg^−1^	1.61	^19^	kgCO_2_-eq kg^−1^	0.192	^19^
Sand	MJ kg^−1^	0.186	^19^	kgCO_2_-eq kg^−1^	0.111	^19^
Steel	MJ kg^−1^	21.3	^19^	kgCO_2_-eq kg^−1^	1.73	^19^
Rice product (whole grains)	MJ kg^−1^	15.2	^36^			
Broken rice, discolored rice, and bran	MJ kg^−1^	9.6	^19^			
Rice husk	MJ kg^−1^	8.7	^19^	kgCO_2_-eq kg^−1^	1.66	^19^
Rice straw	MJ kg^−1^	6.5	^20^			
**Manual labor**						
Operating four-wheel tractor and combine harvester	MJ h^−1^	0.44	^39^			
Operating two-wheel tractor	MJ h^−1^	0.98	^38, 39^			
Manual harvesting, handling, and operating thresher and dryer	MJ h^−1^	0.89	^38, 39^			

^a^For machine production, the energy consumption and GHGE was taken into account through the additional energy (15.2 MJ L^−1^) of fuel used by the machine^44,45^.

The GHGE were accounted for the whole production from cultivation to milling. The yield and grain losses were taken into account through a rice product recovery ratio as shown in Eq. (6).

6 $$GHGE \;(\text{kg} \,{\text{ha}}^{-1})=\frac{\text{G}H{G}_{cultivation}+ GH{G}_{harvest} + GH{G}_{postharvest} }{Product\; recovery\; ratio}$$

where *GHG*_*cultivation*_ for the irrigated rice cultivation in Myanmar was about 2000 and 1200 kgCO_2_-eq ha^−1^ in WS and DS, repectively, as reported in recent research at the same site^40^. GHGE of harvest and post-harvest operations were calculated based on emissions generated during production of harvest and post-harvest equipment, input materials, and fuel consumption during operations. The unit of in-field emissions was per ha while that of off-field emissions was per kg of rice grains. The off-field emission values were therefore translated to attribute for the FU (ha) based on rice yield (kg ha^−1^). Furthermore, the associated unit (kg of rice grains) was considered as the product at the end of the life-cycle boundary, its carbon footprint therefore accounted for the post-harvest losses or product recovery. The recovered rice product (whole grains) was calculated from the grain yield with a consideration of harvest and postharvest losses. The postharvest losses were brokenness (HRR) and discoloration at harvest, drying, storage, milling, and handling. The rice product recovery ratio was calculated based on Eq. (7).

7 $$Product \;recovery\; ratio =\left(1-Los{s}_{harvesting}\right)* HRR*(1-Discoloration)$$

The conversion factors for GHGE of related fuel and power consumption, machine production, and transportation are shown in Table 2. In particular, GHGE from the electric power consumption for drying and milling was translated from Ecoinvent 3 data (version 3.3) for the “rest of the world (ROW)”.

### Cost–benefit analysis

Similar to the energy efficiency analysis, cost-benefits were quantified through the net income value (NIV) (Eq. 8) and net income ratio (NIR) (Eq. 9). NIV accounted for the cost of production and income value (IV) of products and co-products while the NIR was the ratio between NIV and the input cost.

8 $$NIV \left(\frac{\$US}{\text{ha}}\right)=I{V}_{(whole\; rice + broken\; rice + discolored\; rice+bran+husk+ straw)}-(Cos{t}_{cultivation}+{ Cost}_{\left(harvest\; and \;post {\text{-}}harvest\right)}),$$

9 $$NIR=\frac{NIV}{(Cos{t}_{cultivation}+{ Cost}_{\left(harvest \;and \;post{\text{-}}harvest\right)})}$$

The price of rice product was $US 400 per t^1^. Price of discolored rice was assumed to be the same as bran price, which is $US 140 per t^1^. Cost of the cultivation (excluding harvesting and transportation) was about 650 $US ha^−1^ for small-farm irrigated rice production in the Ayeyarwaddy delta of Myanmar^37^. Costs of the post-harvest operations were calculated based on the corresponding depreciation, maintenance, interest, energy consumption, and labor of all related equipment used in the operations from harvesting to milling. The component costs of input materials, labor, and energy included in the analysis were collected based on assessments in Myanmar in 2018 (Table 3).

**Table 3:** Cost and life span of different component costs of input materials, labor, and energy based on assessments conducted in the Ayeyarwady Delta region of Myanmar in 2018.

Component costs	Unit	Value	Life span (years)	Source
Cultivation	$US ha^−1^	650		a
Farmer thresher	$US unit^−1^	500	5	b
Imported thresher	$US unit^−1^	1400	5	b
Combine harvester	$US unit^−1^	30,200	5	b
Flatbed dryer (4 t batch^−1^)	$US unit^−1^	1250	5	b
Granary bag (50 kg paddy)	$US unit^−1^	0.5	2	b
Hermetic super bag (50 kg paddy)	$US unit^−1^	3.0	2	b
Electricity	$US kWh^−1^	0.05		b
Labor	$US h^−1^	0.46		b
Rice product	$US kg^−1^	0.50		c
Discolored rice and bran	$US kg^−1^	0.20		c
Rice husk	$US kg^−1^	0.01		b

a = Soni and Soe^37^, b = assessment in this research, c = World Rice Statistic^1^.

Harvesting loss was used to conduct a sensitivity analysis for NIV and NIR for both the wet and dry seasons. This analysis only applied for the improved post-harvest operations with the flatbed dryer and hermetic storage.

### Statistical analysis and software

Analysis of Variance (ANOVA) Single Factor and Two-Factor with replication and F-Test Two-Sample for Variances tools incorporated in Excel were used to evaluate the effects of the contrasting post-harvest management scenarios on the measured post-harvest losses, energy, and GHGE. The ECOINVENT-3 database (version 3.3)^19^ in association with Cumulative Energy Demand 1.09 method^41^ and the Global Warming Potential—100 years (GWP100a) presented in IPCC 2013^42^, were used to interpret the conversion factors of energy (MJ) embedded and GHGE (CO_2_-eq) from the agronomic inputs and fuel consumption. All these databases and methods (Ecoinvent, Cumulatiive Energy Demand, and IPCC) are incorporated in SIMAPRO version 8.5.0.0^41^.

## Results

### Biomass yield and grain losses

Table 4 shows the grain yield, grain losses and by-products in all three cropping seasons. Yields of paddy at 14% of MC were 2600 to 3100 kg ha^−1^ and 4200 to 5800 kg ha^−1^ for the wet and dry seasons, respectively. Harvesting loss was not significantly different between the treatments in the WS, ranging from 16 to 28% (in weight) with the higher level for FP. However, this factor was significantly different between treatments in the DS. Harvesting loss of the IPRc using a combine harvester was 0.9–1.7% while that of FP was 4.0–9.3%. Stacking harvested rice plants in the field affected significantly the rate of discoloration and HRR. In WS2014, discoloration of grain from FP was 6–8%, which was significantly higher than that of IPR (3.8%). In WS2014, the HRR of the FP samples (range 17–23%) was significantly lower than that of IPR (47%). In the dry seasons, post-harvest losses of IPRc ranged from 3.8 to 6.3% and were significantly lower than that of the FP scenarios (10–17%). Improved post-harvest practice in DS2015 and 2016 significantly increased HRR by 6 to 8%.

**Table 4:** Yield and post-harvest losses for different post-harvest operations across three seasons of rice production in Maubin, Myanmar (WS = wet season; DS = dry season).

Parameters	Unit	WS2014			DS2015		DS2016	
		IPR	FP1w	FP4W	IPRc	FP	IPRc	FP
Yield (crop cut)	kg ha^−1^ (14% MC)	2898 (287.7)	2898 (287.7)	2898 (287.7)	4875 (640.2)	4875 (640.2)	5424 (369.9)	5,424 (369.9)
**Harvesting loss**	%	16.0 (8.72)^a^	28.2 (17.51)^a^	23.63 (15.87)^a^	1.7 (0.28)^d^	9.3 (5.30)^c^	0.9 (0.62)^f^	4.0 (0.49)^e^
Manual cutting and handling	%	13.6 (7.96)	20.8 (16.01)	14.4 (14.48)		6.7 (4.44)		1.8 (0.07)
In-field stacking	%	–	0.3 (0.03)	0.6 (0.29)				
Threshing	%	2.4 (0.76)	7.2 (1.47)	8.7 (1.09)		2.6 (0.86)		2.2 (0.42)
Combine harvesting					1.7 (0.28)		0.9 (0.62)	
Discoloration loss*	%	3.8 (1.13)	6.8 (1.79)	7.9 (1.71)	4.1 (1.49)	5.0 (1.11)	3.3 (0.92)	5.8 (1.22)
Milling recovery	%	64.9 (2.51)^a^	63.4 (3.63)^a^	52.7 (10.63)^b^	63.9 (0.22)^c^	62.6 (0.83)^d^	68.0 (3.76)^e^	64.0 (1.11)^e^
HRR	%	47.2 (6.42)^a^	27.3 (6.21)^b^	17.2 (9.23)^b^	54.8 (2.34)^c^	48.1 (0.83)^d^	64.0 (3.59)^e^	57.5 (1.31)^f^
Milled rice (accounted for physical losses)	kg ha^−1^	1580 (61.2)	1319 (75.4)	1166 (235.3)	3064 (10.3)	2772 (36.9)	3655 (202.2)	3,331 (58.0)
Whole grains	kg ha^−1^	1149 (156.4)	567 (129.3)	380 (204.3)	2628 (111.9)	2130 (36.5)	3437 (193.0)	2,997 (68.0)
Discolored grains	kg ha^−1^	60.5 (17.9)	90.2 (23.6)	91.6 (20.0)	125.3 (45.7)	137.7 (30.7)	119.9 (33.5)	192.8 (40.7)
Broken grains	kg ha^−1^	371 (50.5)	662 (150.7)	694 (372.7)	311 (13.3)	504 (8.6)	98 (5.5)	141 (3.2)
Bran	kg ha^−1^	368 (36.5)	345 (34.3)	605 (60.0)	770 (101.0)	769 (101.0)	644 (43.9)	836 (57.0)
Husk	kg ha^−1^	487 (48.3)	416 (41.3)	443 (43.9)	958 (125.8)	885 (116.2)	1075 (73.3)	1,042 (71.0)
Straw	kg ha^−1^	1449 (144)	1449 (144)	1449 (144)	2438 (320)	2438 (320)	2712 (185)	2,712 (185)

*IPR* improved post-harvest practice, *FP1w* farmer practice with 1 week of stacking harvested rice plants in the field, *FP4w* farmer practice with 4 weeks of stacking harvested rice plants in the field, *IPRc* improved post-harvest practice with combine harvester, *FP* farmer practice without stacking harvested rice plants.

*Discoloration loss caused by the in-field rice plant stacking, drying, and storage; Numbers in the parentheses is the standard deviation; In a row, numbers followed by same letters are not significantly different by F-test at 0.05 level.

### Energy efficiency

Table 5 shows the energy factors including inputs, outputs, NEV, and NER of rice production in the WS and DS. The total input energy of the rice value chain, including production and processing, was in the range of 14–16 and 21–28 GJ ha^−1^ in the WS and DS, respectively. Of which, harvest and post-harvest operations (including machine production depreciation) contributed 16–29% and 25–43% to the total inputs for the wet and dry seasons, respectively. Input energy of the DS production was higher by 30–50% than that of WS for the following reasons: (i) The DS needed higher energy for cultivation (land preparation, water pumping, and agronomic inputs); (ii) The DS had higher rice yield (almost double the WS); the higher yield required higher energy for processing. Input energy of the FP was lower by 15 and 25% than the IPR in the WS and DS, respectively, because of the lower mechanization requirements and lower yields.

**Table 5:** The inputs and outputs, NEV and NER (GJ ha^−1^) of the different rice production treatments in Maubin, Myanmar.

Parameters	Wet season		Dry season	
	IPR	FP	IPR	FP
**Harvesting energy inputs**				
Labor (h ha^−1^)	170.4	141.4	3.0	251.3
Diesel (L ha^−1^)	12.7	7.2	36.0	12.9
**Drying energy inputs**				
Labor (h t^−1^)	2.5	14.0	2.5	14.0
Electric power (kWh t^−1^)	1.3	0.0	1.3	0.0
Rice husk (kg t^−1^)	37.5	0.0	37.5	0.0
**Storage energy inputs**				
Labor (h t^−1^)	1.5	1.5	1.5	1.5
**Milling energy inputs**				
Labor (h t^−1^)	2.0	2.0	2.0	2.0
Electric power (kWh t^−1^)	10.0	10.0	10.0	10.0
Inputs (GJ ha^−1^)	16.88 (4.00)	14.33 (4.00)	28.29 (4.00)	21.23 (4.00)
Cultivation	12.00 (4.00)	12.00 (4.00)	16.00 (4.00)	16.00 (4.00)
Harvest and post-harvest operation (including machine production)	4.88	2.33	12.29	5.23
Outputs (GJ ha^−1^)	34.44 (4.31)	27.95 (6.58)	73.88 (5.23)	68.75 (3.65)
Rice product (whole grains)	17.46 (2.38)	7.20 (2.54)	46.09 (2.32)	38.97 (0.79)
Discolored and broken rice	4.14 (0.66)	7.38 (2.72)	3.14 (0.47)	4.68 (0.40)
Bran	3.53 (0.35)	4.56 (0.45)	6.79 (0.70)	7.70 (0.76)
Husk	4.24 (0.42)	3.74 (0.37)	8.84 (0.87)	8.38 (0.81)
Straw	5.07 (0.50)	5.07 (0.50)	9.01 (0.88)	9.01 (0.88)
NEV (GJ ha^−1^)	17.56 (8.31)	13.62 (10.58)	45.58 (9.23)	47.52 (7.65)
NER	2.04 (1.08)	1.95 (1.65)	2.61 (1.31)	3.23 (0.91)

Numbers in parentheses is the standard deviation.

*IPR* improved post-harvest practice, *FP* farmer practice.

NEV, in the ranges of 14–18 and 46–48 GJ ha^−1^ in the WS and DS, respectively, was not significantly different between FP and IPR in each season. However, this factor was about 3 times higher in the DS than in the WS because of the substantially higher yield and lower grain losses. The lower loss of the IPR compensated for the higher inputs so that the NEV of IPR and FP did not differ significantly. Consequently, NER, with a mean ranging from 1.95 to 3.23, was not signficantly different between FP and IPR, and between the WS and DS.

### Greenhouse gas emissions

Table 6 shows the GHGE of rice cultivation, harvest, and post-harvest production for IPR and FP in the WS and DS. There was no significant difference of GHGE between FP and IPR. However, GHGE from rice production in the WS was 5.3–5.7 Mg CO_2_-eq ha^−1^; almost double that of the DS because of the higher emissions during cultivativaltion and higher grain loss. The total GHGE consisted of 70–90% from cultivation, 5–15% from harvest and 5–10% from post-harvest operations. Hidden under the rice product recovery ratio, grain losses caused an increase of GHGE (per ha of rice production) by about 30–50%.

**Table 6:** GHG emissions (kg CO_2_-eq ha^−1^) of the different rice production treatments in Maubin, Myanmar.

Parameters	Wet season		Dry season	
	IPR	FP	IPR	FP
Cultivation	3823 (956)	4900 (225)	1888 (629)	2104 (701)
Harvesting	698 (30.4)	536 (77.5)	88 (2.7)	470 (7.8)
Drying	582 (25.3)	64 (9.2)	48 (1.5)	77 (1.3)
Storage	44 (1.9)	43 (6.2)	4 (0.1)	51 (0.8)
Milling	116 (5.1)	149 (21.6)	10 (0.3)	1793.0)
Paddy transportation (15 km)	33 (1.4)	43 (6.2)	3 (0.1)	51 (0.8)
Total	5297 (1020)	5734 (1346)	2039 (634)	2933 (715)

Numbers in parentheses is the standard deviation.

*IPR* improved post-harvest practice, FP farmer practice.

### Cost of post-harvest losses and operations

Table 7 shows the cost components, NIV, and NIR of rice production for the IPR and FP in the WS and DS. The total input cost of the rice value chain, including production and processing, was in the range of 744–769 and 832–899 $US ha^−1^ in the WS and DS, respectively. Of which, harvest and post-harvest operations (including depreciation, maintenance, fuel, power, and labor) contributed 13–15% and 22–28% to the total inputs for the wet and dry seasons, respectively. Production costs for the DS was higher by 8–17% than the WS because of the higher yield obtained in the DS.

**Table 7:** Cost-benefits of the different rice production treatments in Maubin, Myanmar.

Parameters	Wet season		Dry season	
	IPR	FP	IPR	FP
Inputs ($US ha^−1^)	768.7	744.0	831.9	898.8
Cultivation	650.0	650.0	650.0	650.0
Harvest and post-harvest operation^a^	118.7	94.0	181.9	248.8
Outputs ($US ha^−1^)	850.4 (33.00)	693.2 (81.01)	1794.6 (56.76)	1646.2 (29.73)
Milled rice	759.9 (21.65)	575.7 (66.80)	1618.5 (33.35)	1443.1 (5.86)
Discolored rice and bran	85.6 (10.87)	113.2 (13.79)	165.9 (22.42)	193.5 (22.94)
Husk	4.9 (0.48)	4.3 (0.43)	10.2 (1.00)	9.6 (0.94)
NIV ($US ha^−1^)	81.7 (33.00)	− 50.8 (81.01)	962.7 (56.76)	747.5 (29.73)
NIR	0.1 (0.04)		1.2 (0.07)	0.8 (0.03)

Numbers in parentheses is the standard deviation.

*IPR* improved post-harvest practice, *FP* farmer practice, *NIV* net income value, *NIR* net income ratio.

^a^Including machine depreciation and maintenance, fuel, power, and labor.

NIV was considerably different between the DS and WS. The DS yield double that of the WS and there were significantly lower losses in the DS. The NIV in the wet season was 748–963 $US ha^−1^, whereas the WS had almost no net profit. These findings are consistent with feedback from farmers who reported that they had little profit from rice production in the WS except for their labor-recovery costs. Overal, NIR of IPR using combine harvester in the dry season was higher by 30–40% than that of FP.

**Figure 3:**
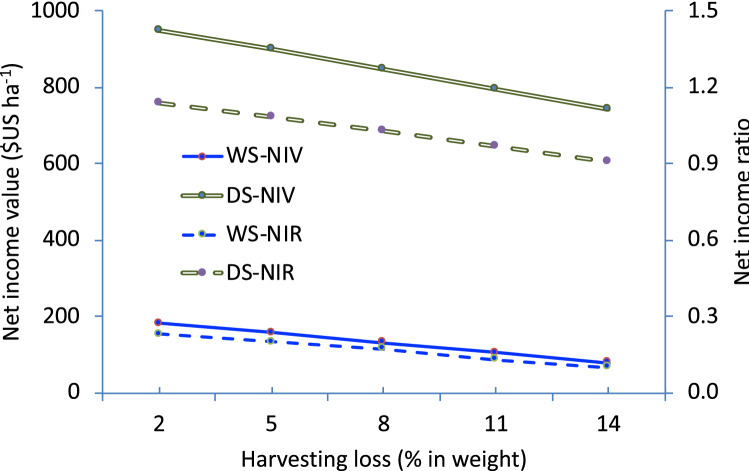
Effects of harvesting loss on NIV ($US ha^−1^) and NIR for WS and DS.

Figure 3 shows the linear effects of harvesting loss on NIV and NIR for both the WS and DS. When harvesting losses from improved practices were reduced from 14 to 2% of production, the NIV increased from 78 to 182 $US ha^−1^ and 744 to 950 $US ha^−1^ for the WS and DS, respectively. Consequently, NIR increased from 0.10 to 0.25 and 0.91 to 1.14 for the WS and DS, respectively.

## Discussion

**Figure 4:**
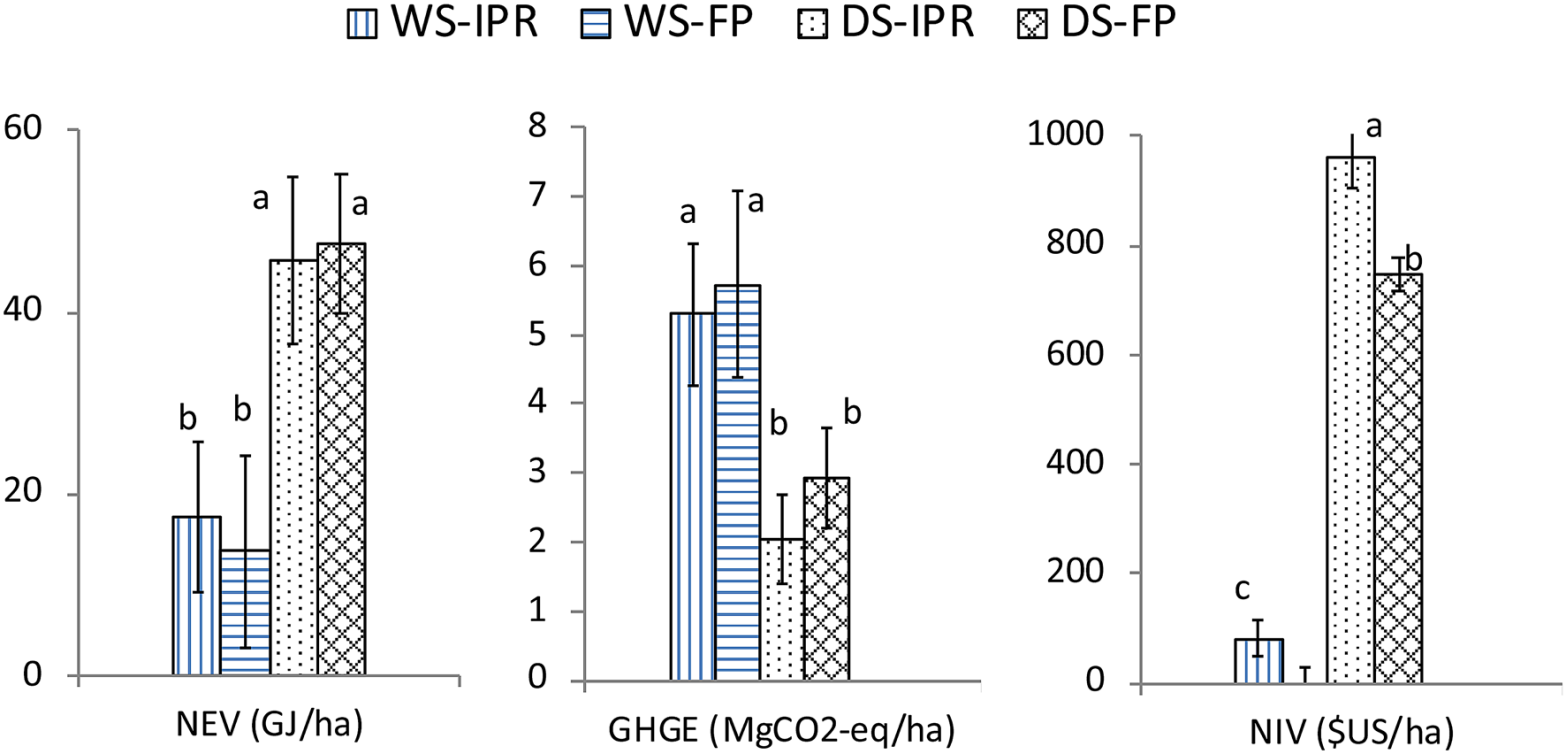
Energy balance, GHGE, and cost-benefits of rice production with different harvest and post-harvest practices. *WS* wet season, *DS* dry season, *IPR* improved practice, *FP* farmer practice; In a factor (i.e. NEV, GHGE, and NIV), numbers followed by same letters are not significantly different by F-test at 0.05 level.

Improved post-harvest practices using a combine harvester, flatbed dryer, and hermetic storage bags significantly reduced losses and increased profit for smallholder farmers of lowland rice in the Ayeyarwady region of Myanmar (see Fig. 4). Combine harvesting helped to significantly reduce harvesting loss in a range of 3–7% (by weight of the rice products) in DS2015 and DS2016, and post-harvest losses and production costs of IPR were significantly reduced compared to those of current farmer practices. The total energy consumption was not significantly higher than FP in the dry season and was significantly lower that all other practices not using combine harvesters in the wet season. This is a robust finding given that the energy consumption to produce the equipment and from fossil fuel use were considered. Similarly, GHGE of IPR was not significantly higher than that of FP. Lower grain loss and higher rice production recovery ratio led to 40–60% lower GHGE in the DS than in the WS.

An investigation conducted by the FAO^2^ revealed that post-harvest losses can reach up to 30%. The current study revealed that post-harvest losses in Tar Pat Village, Maubin, Myanmar reached up to 50% with current farmers’ practices. Post-harvest losses impact substantially on the profitability of rice production, and on the energy efficiency and GHGE of this lowland rice system, which is demonstrated in the LCA conducted in the current study.

An interview-based LCA study on rice production in Taiwan reported in Hung-Chun and Yasuhiro^43^, indicated that energy consumption and GHGE from drying and refining were 2 MJ kg^−1^ and 0.1 kg CO_2_ kg^−1^ of milled rice, respectively. In the current study, excluding the post-harvest losses, our findings are comparable. The energy consumed and GHGE during the processes from drying to milling were 1.7–3.8 MJ kg^−1^ and 0.1–0.35 kg CO_2_ kg^−1^ of milled rice. The differences are most probably due to differences in rice production practices, rice germplasm, and technologies used in each system.

To achieve optimal outcomes in mechanized systems it is crucial that efficient and effective operations and practices are followed. In the field study in the Ayeyarwaddy delta, the timing of harvest and the harvesting operations with combine harvesters were managed with care using experienced operators. Drying of harvested grain was undertaken within 24 h of harvest to protect grain quality and ensured that weather conditions did not degrade the grain. Additionally, care was taken in ensuring that harvested grain was stored correctly to reduce risks associated with storage pests, and milling was undertaken with accurately calibrated rice milling equipment. If capacity, knowledge and skills are not adequate, losses in mechanized systems are likely to be higher than in the current study.

The cost analyses for the different seasons varied depending on labor, fuel, and equipment rental, and are specific for the study area. However, the significant difference in cost between IPR and FP was largely the result of the high costs associated with post-harvest losses, which were 30–50% in the FP scenario. Unlike labour, fuel and equipment rental, the parameters for cost analysis of energy efficiency and GHGE were calculated based on conversion factors provided in the global databases ECOINVENT^19^ and IPCC^42^. Thus, these factors are not greatly affected by the local context such as price of labor, rice, and fuel.

This research provided primary quantitative data based on field experiments for improved harvest and post-harvest practices in the fields of farmers. Nevertheless, there are some limitations to the interpretation of the findings. Firstly, secondary data was used for the cultivation stages. Second, the experiment plots were limited in size with 300–500 m^2^ per plot. Although, this would mainly affect estimates of the harvesting loss, and was already reflected in the sensitivity analysis of the cost-benefits. Third, the energy associated with the rice straw was accounted for but for GHG emissions estimates for in-field emmisions were based on data from a previous study^40^. Finally, conversion factors for input materials and power consumption were cited from the Ecoinvent 3 database, based on the general region “rest of the world (RoW)” that including Myanmar.

The findings of the current study have important implications for farmers, machinery service providers, and policy makers. The findings provide objective evidence for farmers to make informed decisions on selecting cost-effective mechanized post-harvest practices for their rice production. The information from this study will enable service providers to develop effective business models for mechanized operations based on the realized economic and environmental benefits. Evidence that sustainable cost-saving benefits from mechanization can be achieved without increasing GHGE, provides valuable information for policy makers involved in formulating evidence-based mechanization policies.

## Conclusions

The study demonstrates that the IPR with higher mechanization increased the net income by 30–50% compared with FP. Despite using additional energy for machine manufacturing and fuel consumption, the IPR significantly reduced postharvest losses and did not increase the total life-cycle enegy and GHGE. Energy requirements to purchase and produce equipment in mechanized harvest and post-harvest systems can be offset by reduced production losses when compared to traditional post-harvest operations. Combine harvesting helped to significantly reduce harvesting loss in a range of 3–7% (by weight of the rice products). Improved post-harvest management practices with flatbed dryer, hermetic storage helped to reduce the discoloration 3–4% and increase the HRR by 20–30% (by weight of rice product). With a higher yield, lower grain loss, and higher rice product recovery ratio, NEV and NIV of DS were higher by 30–50% than those of WS; and GHGE of the DS was lower by 40–60% than that of WS. Similar LCA studies in other rice producing countries will strengthen the development of recommendations for improved post-harvest management of rice.

## Abbreviations

CO_2_-eq: Carbon dioxide equivalent
DS: Dry season
EV: Energy value
FP: Farmer practice
FU: Functional unit
GHGE: Greenhouse gas emissions
IPR: Improved practice
IPRc: Improved practice with combine harvester
LCA: Life cycle assessment
NEV: Net energy value
NER: Net energy ratio
NIV: Net income value
NIR: Net income ratio
SEA: Southeast Asian countries
WS: Wet season
h: Hour
ha: Hectare
HRR: Head rice recovery
kg: Kilogram
kWh: Kilowatt * hour
L: Liter
MC: Moisture content in wet basis
MJ: Mega joules
MET: Metabolic equivalent of task
ton: Mega gram
tkm: Ton * km
